# α-Mangostin Nanoparticles Cytotoxicity and Cell Death Modalities in Breast Cancer Cell Lines

**DOI:** 10.3390/molecules26175119

**Published:** 2021-08-24

**Authors:** Yedi Herdiana, Nasrul Wathoni, Shaharum Shamsuddin, Muchtaridi Muchtaridi

**Affiliations:** 1 Department of Pharmaceutics and Pharmaceutical Technology, Faculty of Pharmacy, Universitas Padjadjaran, Sumedang 45363, Indonesia; nasrul@unpad.ac.id; 2 School of Health Sciences, Universiti Sains Malaysia, Kubang Kerian 16150, Kelantan, Malaysia; shaharum1@usm.my; 3 Nanobiotech Research Initiative, Institute for Research in Molecular Medicine (INFORMM), Universiti Sains Malaysia, Gelugor 11800, Penang, Malaysia; 4 USM-RIKEN Interdisciplinary Collaboration on Advanced Sciences (URICAS), Universiti Sains Malaysia, Gelugor 11800, Penang, Malaysia; 5 Department of Pharmaceutical Analysis and Medicinal Chemistry, Faculty of Pharmacy, Universitas Padjadjaran, Sumedang 45363, Indonesia

**Keywords:** AMG, breast cancer, cell death, apoptosis, cytotoxicity, nanotechnology

## Abstract

α-Mangostin (AMG) is a potent anticancer xanthone that was discovered in mangosteen (*Garcinia mangostana* Linn.). AMG possesses the highest opportunity for chemopreventive and chemotherapeutic therapy. AMG inhibits every step in the process of carcinogenesis. AMG suppressed multiple breast cancer (BC) cell proliferation and apoptosis by decreasing the creation of cancerous compounds. Accumulating BC abnormalities and their associated molecular signaling pathways promotes novel treatment strategies. Chemotherapy is a commonly used treatment; due to the possibility of unpleasant side effects and multidrug resistance, there has been substantial progress in searching for alternative solutions, including the use of plant-derived natural chemicals. Due to the limitations of conventional cancer therapy, nanotechnology provides hope for effective and efficient cancer diagnosis and treatment. Nanotechnology enables the delivery of nanoparticles and increased solubility of drugs and drug targeting, resulting in increased cytotoxicity and cell death during BC treatment. This review summarizes the progress and development of AMG’s cytotoxicity and the mechanism of death BC cells. The combination of natural medicine and nanotechnology into a synergistic capital will provide various benefits. This information will aid in the development of AMG nanoparticle preparations and may open up new avenues for discovering an effective BC treatment.

## 1. Introduction

The use of chemotherapy is not selective for cancer tissue, and thus it damages normal tissue. Surgery, chemotherapy, and radiotherapy have become the mainstay of cancer treatment. The problems that arise are metastasis, drug resistance, toxicity, and unwanted side effects [1,2,3,4]. Effective, efficient, cost-effective, and biocompatible alternative medicine is urgently needed [5].

Anticancer drugs derived from natural ingredients are a trendy choice; they emerged as promising candidates for anticancer therapy by targeting multiple signaling pathways [6]. Many of them are designed and developed from natural plants, showing good trends with new anticancer mechanisms, low toxicity, and inhibiting cancer stem cell (CSC) stems.

Currently, AMG is the most potent chemopreventive agent and chemotherapy. AMG can prevent carcinogenesis at all stages (cell division, cell proliferation, apoptosis, inflammation, and metastasis). AMG inhibits kinases, cyclooxygenase, ribonucleotide reductase, and DNA polymerase in tumor cells [3,7,8,9,10]. Considerable evidence from in vitro and in vivo studies has confirmed that AMG inhibits various tumor cell proliferations by modulating multiple targets and signal transduction pathways [8]. AMG has better cytotoxicity and selectivity against cancer cells. An exposure cytotoxic antitumor response can lead to tumor cell death [11]. Cell death that occurs can be apoptosis, autophagy, or necrosis [12].

The NPs enable delivery to cells and damaged intracellular organelles [13]. NPs have distinct biological and toxicological effects. Small-sized NPs can be endocytosed by cells, enhancing cytotoxicity [14]. Molecular targeted anticancer drugs and NPs’ surface features affect their high precision in treating cancer [15,16].

This review collects and discusses a better understanding of breast cancer’s molecular aspect and AMG’s molecular cytotoxic mechanisms; this knowledge can help provide a better NPs preparation drug delivery system. Combining natural products and the application of NPs could significantly improve conventional therapy with their biocompatibility, as they have no adverse consequences.

## 2. Chemical Characteristics of AMG

**Figure 1:**
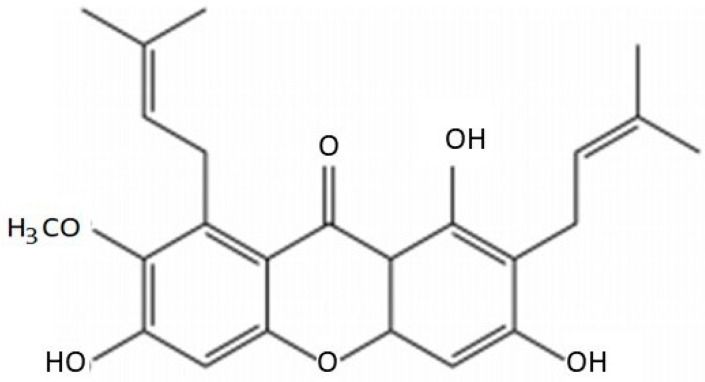
Composition of α-mangostin.

The chemical content of mangosteen peel contains vitamins, minerals, phosphorus, iron, and natural polyphenols such as xanthones [17]. The secondary metabolites of xanthones comprise the class of oxygenated heterocycles [18]. AMG is a metabolite of 1,3,6,7-tetrahydroxy-2,8-di (3-methyl-2-butenyl) xanthones (Figure 1). The molecular formula of AMG is C_24_H_22_O_6_, with a molecular weight of 410.46 and a melting point of 180–182 °C [19]. AMG formulations often require large solubilizer concentrations because of their poor water solubility, thus limiting their bioavailability and usage in certain therapeutic applications [20].

## 3. The Activity of AMG in Breast Cancer

Over 200 novel chemical compounds have been licensed for use in the battle against cancer in the last 50 years, with about 50% being molecules of unaltered natural products [21]. Small organic molecules of secondary metabolites have played an essential role in inhibiting proliferation-induced apoptosis or other modifiable mechanisms [22,23,24]. Natural products and their derivatives have been recognized for many years by the pharmaceutical industry [25,26].

### 3.1. Molecular Development of Breast Cancer

**Figure 2:**
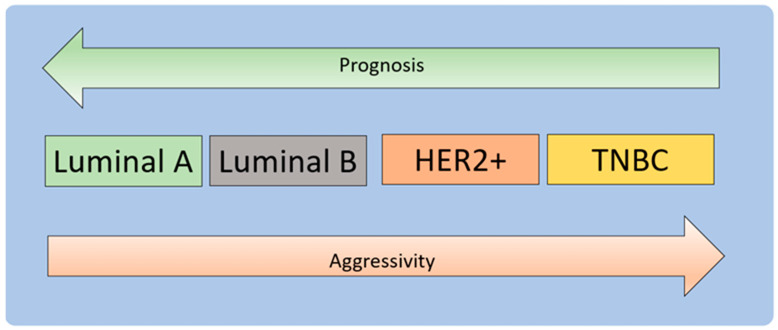
Molecular subtypes of breast carcinoma. BC can be based on hormonal receptors, and HER2 status is divided into luminal types A and B, HER2+, and TNBC [30]. Luminal A and luminal B represent [ER+|PR+]HER2 (tumors with positive ER or PR and negative HER2) and [ER+|PR+]HER2+ subtypes (tumors with positive ER or PR and positive HER2). Luminal A tumors have higher expression of ER-associated genes and lower expression of proliferative genes than luminal B cancers [31,32]. Luminal B tumors tend to have a higher grade than luminal A tumors. The luminal subtype generally carries a good prognosis, and luminal B tumors have a much poorer prognosis than the luminal A subtype [33]. The basal subtype comprises ER-PR-HER2- (triple-negative) tumors with an expression profile that mimics basal epithelial cells from other parts of the body and normal breast myoepithelial cells [34]. This subtype has a low expression of hormone receptors and HER2 and high expression of basal markers and proliferation-associated genes. The tumor has a difficult prognosis, an aggressive clinical course, and currently lacks a standard targeted form of systemic therapy. The pattern of metastases tends to be visceral (excluding bone) and is less likely to involve lymph nodes. Tumors of this class tend to show rapid growth [35].

BC is one of the most well-known cancer types, having various pathological subtypes and clinical outcomes. BC may be caused by a mutation that results from the aging process and risk factors associated with a healthy lifestyle [27]. The most promising strategy for cancer treatment is to disrupt the three phases of carcinogenesis—initiation, promotion, and progression—and alter the carcinogenesis signaling pathways [4]. BC treatment may be quite successful, mainly when the illness is detected early and has a favorable prognosis (Figure 2) [28,29].

Cancer develops through a series of molecular processes, namely mutations in DNA molecules that code for proteins that initiate cell division, proliferation, and growth. As a result, damage to DNA or proteins that regulate the cell cycle can result in uncontrolled cell division and proliferation, referred to as cancer. Cancer causes dysregulation of apoptosis, proliferation, angiogenesis, and metastasis [4].

BC is molecularly classified into estrogen receptor (ER) and progesterone receptor (PR) expression and human epidermal growth factor receptor (HER2) amplification [4]. Approximately 60–70% of early BC patients are hormone-sensitive, showing positive estrogen receptor (ER+), positive progesterone receptor (PR+), or both. About 15–20% of BC patients exhibit a triple-negative phenotype due to the absence of ER, PR, and HER2 amplification. Approximately 20% of BC cases show HER2 overexpression, resulting in aggressive disease and reduced survival [36,37].

BRCA1 is a human tumor-suppressor gene responsible for repairing damaged DNA or destroying cells if the DNA cannot be repaired. BC 1 (BCA1) and BC 2 (BCA2) are essential for genome stability. Mutations in genes give the risk of BC up to 15–20 times [38,39,40].

The tumor suppressor TP53 gene encodes the protein p53, which regulates the cell cycle, induces apoptosis, maintains genome integrity, and prevents tumor development. Mutations in the TP53 gene increase the risk of BC [4]. When stress occurs, phosphorylation of p53 interferes with binding to protein MDM2, resulting in p53 accumulation and subsequent transcription of several genes, including the gene encoding the protein cyclin-dependent kinase inhibitor (CKI) p21. P21 interacts with and inactivates the G1/S-Cdk complex, reprogramming cells to G1 for DNA repair [41,42].

BC treatment protects genomic stability by avoiding DNA damage, delaying the cell cycle or inducing apoptotic cell death, and influencing aberrant cell proliferation pathways [4]. Chemotherapy can inactivate normal cell p53 and induce apoptosis in regularly developing cells/tissues, such as bone marrow, lymphoid organs, hair follicles, and small intestinal epithelium [2,43,44].

### 3.2. Mechanisms of Molecules Drug Resistance (MDR)

Overexpression of MDR1 is one of the main reasons for multidrug resistance to chemotherapeutic drugs [45]. The expression of P-glycoprotein encoded by the multidrug resistance (*MDR1*) gene is associated with the emergence of the MDR phenotype in cancer cells [46]. P-glycoprotein (P-gp) is a well-identified membrane transporter with the capability to efflux drug molecules out of the cancer cell, leading to the reduced efficiency of chemotherapy [47]. MDR mechanisms fall into different categories: (1) enhance the efflux of drugs across membrane carriers, using ATP binding cassette (ABC) carriers as major carriers; (2) diminished uptake of drugs via influx carriers, such as solvent carriers; (3) enhanced metabolic drugs, including the removal of S-transferase glutathione and P450 cytochrome enzyme; and (4) inhibiting of apoptotic pathways [48,49,50,51].

### 3.3. Cytotoxicity

Cytotoxicity refers to a poisonous agent’s toxic effect on cells. Cytotoxic agents inhibit cell growth and can occasionally result in cell death [52]. Exposure of cells to cytotoxic substances can result in various cell-death consequences [53,54,55]. Following that, cells may initiate a program of programmed cell death (apoptosis) or necrosis [56,57]. Cells that undergo fast necrosis in vitro lack the time and energy necessary to activate apoptotic pathways and do not exhibit apoptotic markers. Secondary necrosis occurs when apoptotic cells in culture experience apoptosis [58]. The causes of cytotoxicity are divided into the following: (1) Chemical cytotoxic agents—the primary goal for cytotoxic drugs, or cytostatic agents, is to prevent the growth of cancer cells, often accomplished by targeting processes that directly disrupt DNA replication or transcription interfering with critical cell processes during mitosis [59]. (2) Biological cytotoxic agents—biological cytotoxic agents are commonly defined as harmful compounds produced from viruses, bacteria, fungus, plants, or animals. The most well-known compounds in this category are bacterial endo-/exotoxins and antibiotics [58,60]. (3) Physical cytotoxic agents—heat, ultrasonic vibrations, and radiation are all examples of physical agents that are cytotoxic. It has been discovered that using ultrasonic microbubbles enhances the cytotoxicity of chemotherapy medicines on tumor cells. Numerous research studies on radiation’s cytotoxicity may be found in the literature [59].

### 3.4. Mechanisms of Cell Death

Cell death is a type of cellular suicide that is regulated by an intracellular program and acts as an innate method to maintain the balance of cell survival. In humans, cells undergo three stages of aging, namely (1) proliferation for a certain number of cycles; (2) loss of replicative ability, and then inactivity for a certain period of time; and (3) then they dying and death [61]. Many stimuli cause cell death, and the mode of cell death will follow one of two general patterns. The first is necrosis; oncotic necrosis is most often the result of a severe metabolic disorder and is characterized by cellular swelling, leading to the rupture of the plasma membrane with the release of intracellular contents. The second pattern is apoptosis, a form of programmed cell death. Apoptosis causes regular resorption of individual cells initiated by a well-defined pathway involving the activation of proteases called caspases [62].

Apoptosis is essential for maintaining tissue homeostasis in balance with cell proliferation and death. Unwanted, old, damaged, and altered cells of an organism must be removed by apoptosis [63]. Apoptosis also protects cells in disease states or harmful chemicals, for example, in immunological reactions [64]. Moreover, p53 is a cellular stress sensor and a major pathway activator. The extrinsic pathway begins outside the cell when it is determined in the extracellular environment that the cell must be killed [65,66]. Clearing damaged organelles is necessary for autophagy to support cell survival. Cell death and cell survival are represented by apoptosis. Induction of caspases or degradation of endogenous apoptosis inhibitors can be preceded or enhanced by autophagy [67]

In contrast to necrotic cell death, which usually occurs due to adenosine triphosphate (ATP) depletion, apoptosis is an ATP-requiring process. Different organelles (plasma membrane, mitochondria, nucleus, endoplasmic reticulum, and lysosomes) elicit cell-death signals. In particular, mitochondrial permeabilization and dysfunction usually develop in necrosis and apoptosis. In some cases, apoptosis and necrosis share a signaling pathway, resulting in programmed necrosis called necroptosis. In this way, apoptosis and necrosis may represent extreme endpoints on the phenotypic continuum of lost cell viability [68].

## 4. Molecular Cell Death Mechanisms of AMG in Cancer Cells

### 4.1. In Vitro Effect of AMG in Breast Cancer

AMG extracted from the pericarp of *G. mangostana* L. proved pharmacologically in vitro and in vivo associated with cancer (Table 1) [69].

**Figure 3:**
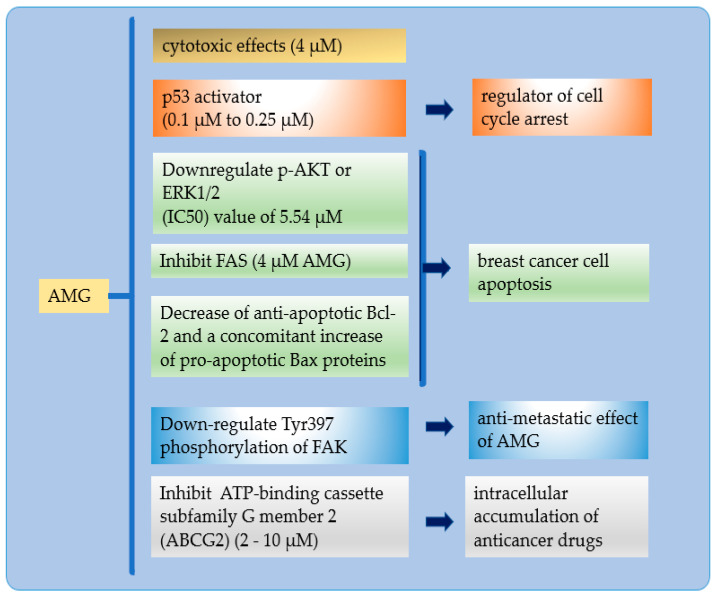
Hallmark mechanism of AMG in BC.

The inhibition of ER stress and autophagy while treating cells with AMG significantly enhanced the apoptosis of cells [75]. AMG targets multiple signaling pathways involved in cell-cycle modulation and apoptosis [78]. AMG’s effects include apoptosis, cell growth suppression, proliferation, cell-cycle arrest promotion, and metastases [75]. The shown mechanism was apoptosis and was identical to time-dependent activation, including DNA fragmentation and caspase-3 cleavage (Figure 3).

The hallmark mechanism of AMG in BC: (a) AMG has excellent cytotoxicity in small doses; (b) AMG inhibits p53 binding to the mouse double minute (2MDM2), resulting in a cell cycle for low-level stressors, reducing tumor formation; (c) enhanced BC apoptosis by suppression of the phosphatidylinositol 3-kinase (PI3K)/protein kinase B (AKT) signaling pathway, suppresses folic acid synthesis (FAS) expression, and inhibits anti-apoptosis of the B-cell lymphoma-2 (BCL-2) family of proteins and increase pro-apoptotic Bax protein); (d) inhibits tumor development and metastasis by FAK activation and is mediated by autophosphorylation at tyrosine-397; and (e) enhanced intracellular accumulation of anticancer drugs by inhibition the ATP-binding cassette transporter G2 (ABCG2).

Activation of the p53 pathway reduces tumor formation. This p53 mutation occurs due to a single amino acid substitution in the middle region of the p53 protein. AMG inhibits p53 binding to MDM2, resulting in a cell cycle for low-level stressors [2]. Restoration of p53 activity by inhibiting p53−MDM2 interactions is a therapeutic strategy and focuses on significant cancer-drug discovery [79].

Inhibition of FAS forms the basis for AMG as a bright candidate for anticancer. FAS expression is the most common molecular change in breast malignancy and targets BC therapy [76]. Suppression of the PI3K/AKT signaling pathway suppresses intracellular lipid accumulation in adipocytes, differentiating and stimulating lipolysis in mature adipocytes [79].

The BCL-2 family of proteins is responsible for mitochondrial apoptosis [80]. An explosion of BC cells with AMG will decrease anti-apoptotic Bcl-2. The pro- versus anti-apoptotic balance of BCL-2 protein is a regulator of programmed cell death. When out of balance, it will block apoptosis and facilitate tumor development and resistance to cancer therapy [80,81].

AMG can modulate tumor migration and invasion by downregulating MMP-2, MMP-9, and urokinase-type plasminogen activator (μPA) by suppressing ERK-mediated pathways and reducing AP-1 and NK-kB binding in BC [17,82]. AMG was able to inhibit tumor development and metastasis in a mouse model of breast cancer. AMG decreases levels of phospho-Aktthreonine 308 (Thr308) in mammary carcinoma tissue in vivo [71]. FAK activation is mediated by autophosphorylation at tyrosine-397 [76]. AMG exposure to MDA-MB-231 decreased cell adhesion, which did not stimulate cell migration [9].

The G2 ATP-binding cassette transporter is involved in clinical multidrug resistance (MDR). As an efflux pump, ABCG2 excretes a variety of endogenous and exogenous substrates [83]. Efficiency reduces intracellular drug accumulation [84,85]. AMG effectively and selectively inhibited ABCG2-mediated drug transport and reversed MDR in ABCG2-expressed MDR cancer cells. AMG binds to the ABCG2 substrate-binding pocket and competitively attenuates the ABCG2 transport function [49].

### 4.2. In Vivo Effect of AMG in Breast Cancer

The treatment with 20 mg/kg/day AMG resulted in prolonged survival rates and increased inhibition of tumor growth and lymph-node metastasis in metastatic mammary carcinoma mice [86]. AMG could inhibit the growth of Cholangiocarcinoma Cells and Allografts (CCAs), i.e., reduce tumor mass (weight and size) and alter CCA pathology [87]. Attempts have been made to forecast the potential of therapeutic candidates; one such strategy is in vitro/in vivo correlation (IVIVC) [88]. In this regard, IVIVC is critical in pharmaceutical science to create dosage formulations. Improved in vitro/in vivo correlations (IVIVCs) for drug products may allow more reliance on in vitro data when making novel drugs, resulting in expedited pharma product development and lower research and development expenditures.

## 5. Nanotechnology of AMG in Breast Cancer

Bioactive secondary metabolites, such as flavonoids, terpenoids, alkaloids, tannins, and others, have been recognized as potential medical resources [89,90]. As shown in Section 3 and Section 4, AMG has high cytotoxicity. However, AMG has a problem in solubility, thus resulting in having poor target selectivity in the human body. Furthermore, effort is needed to increase the solubility, selectivity, and efficacy of α-mangostin as a new drug candidate in clinical therapy.

Nanotechnology is predicted to alleviate some of the drawbacks of current therapies [91,92]. Nanotechnology produces materials of various types at the nanoscale level. NPs are a broad class of materials that includes particulate matter, with one dimension less than 100 nm at least [16]. The physical and chemical properties of nanosized materials differ substantially from the properties of the same material in bulk [93,94]

Nanotechnology has emerged as a great strategy to overcome the challenges of systemic toxicity, low solubility, poor bioavailability, high plasma protein binding, non-target drug accumulation, poor drug-receptor/tissue interactions, low penetration, and internalization into target tissues, as well as sub-therapeutic pharmacologic responses. Its core properties and surface functionalization facilitate the delivery of therapeutic drugs or molecules and enhance therapeutic efficacy [95,96,97,98].

NPs attract great attention as multifunctional nanocarriers in DDSs, by combining the specific targeting with the transport and release of a contrast agent [99,100]. Several chemotherapeutics have been encapsulated in NP delivery systems to increase antitumor efficacy, inhibiting metastases, and decrease the effective dose and side effects [101]. Cancer drug-targeting strategies can be further advanced by developing DDSs, using stimuli-responsive nanomaterials, which can be performed based on endogenous and exogenous stimulant factors [102], including passive or active drug targeting in tumor tissues and improved intracellular penetration [96].

The high entrapment efficiency can be attributed to the hydrogen bonding interactions between xanthones and carrier molecules. The substantial increase in xanthone solubility, endocytotic uptake of cationic NPs, and intracellular xanthone delivery may provide a novel drug delivery system for treating and preventing gastrointestinal tract tumors, especially small-intestine and colon tumors [103].

AMG NPs are used to increase solubility in water, provide a controlled release, and create targeted delivery systems. The forms of NPs for AMG are polymeric NPs, nano micelles, liposomes, solid lipid NPs, nanofibers, and nanoemulsions. Advances in nanotechnology have provided a new way of improving drug delivery systems (DDS) for hydrophobic drugs. The poly(ethylene glycol)–poly(e-caprolactone)–poly(ethylene glycol) triblock copolymer (PEG–PCL–PEG, PECE) is a type of biodegradable and amphiphilic polymer micellar that is formed by the self-assembly of two hydrophilic PEGs and hydrophobic PCL, resulting in a hydrophobic inner core and a hydrophilic outer shell [104].

Wathoni et al. showed that nanomicelle modification increased the solubility of AMG by more than 10,000-fold. The formulation of NPs affects their biopharmaceutical, pharmacokinetic, and pharmacodynamic aspects. In addition, polymeric NPs provide targeted delivery and significantly enhance AMG biodistribution to specific organs [105].

Miftahul et al. showed electrospun PVP nanofibers of mangosteen peel extract, increased antioxidant properties, and solubility of AMG due to increased surface area, using a highly water-soluble excipient, PVP, as the main base for NPs increases drug solubility mediated by hydrogen bonding interactions between excipients and water molecules [106]. Wathoni et al. demonstrated the effect of enhancing properties and compounds of AMG by using the AMG-Chitosan/Carrageenan NP system. These NPs impact the physicochemical properties of AMG, improve the poor water solubility profile, and increase cytotoxicity [107].

In some cases, nanocarriers are combined with a targeting mediator to obtain a drug delivery system targeted by NPs to a specific target. Pharm et al. showed that AMG-loaded crosslinked silk-fibroin-based NPs maintain AMG’s apoptotic effect while exhibiting more significant cytotoxicity than the free drug [108]. Bonafe et al. demonstrated that AMG loaded in CD44 Thioaptamer-tagged NPs reduces the number of BC cancer cells [7]. NPs that mediate passive or active target delivery are generally prepared with a high affinity for the target and low affinity for normal cells [105].

NPs can passively accumulate in the cancer cells because of the enhanced permeability and retention (EPR) effect [109,110,111]. Furthermore, the capability of surface modifying NPs allows for active targeted cells by combining monoclonal antibodies and tumor-specific ligands with the NP [112]. With the advantages of NPs, the NP drug delivery system can be enhanced the cytotoxicity of free AMG. Various benefits of NPs have been described above, but their use in delivering AMG in BC therapy has not been widely studied.

Through cytotoxic stress, NPs can impair normal cellular function and are responsible for membrane damage. NPs can be used in the therapy of BC by increasing the following: (1) technologies to strengthen the solubility and stability of anticancer drugs; (2) passive and active targeting used by nano-based modalities to selectively target malignant tissues/cells; (3) distribution of various drugs to help decrease drug resistance; (4) nano-based vehicles with controlled-release techniques for medication; (5) usage of nanoformulation, which is dependent on stimulus responsiveness; (6) drug efflux pathways can be blocked by nano-based vehicles; and (7) delivery of different medications and, thus, aims to minimize drug resistance [113].

Understanding the mechanism of nanotoxicity is critical to the design of safe NP-based systems. On the other hand, there is emerging evidence that the cytotoxic potential of NPs can be exploited in cancer treatment. NPs may have therapeutic value and modulate apoptosis, necrosis, necroptosis, and autophagy [61]. Cancer nanomedicine can be designed to remove cancer, and nanomaterials can cause micro gaps in the endothelial walls of blood vessels. The possibility of endothelial leakage caused by nanomaterials must be considered while developing future nanomedicines, particularly those used to treat cancer [114]. The delivery of AMG with can be achieved with a nanoparticle system, in accordance with improvements in pharmacokinetic properties, drug release, and drug targeting [105].

Nanotechnology has succeeded in increasing bioavailability and distribution, maintaining the integrity of compounds, and increasing the biological activity of cancer drugs. AMG-NPs inhibited pancreatic cancer cell growth and CSC characteristics in vitro and tumor growth, development, and metastasis in mice. AMG NPs provide clinical significance in cancer and other diseases. Gold-encapsulated AMG/polyethyleneimine/cyclodextrin (AuNPs/PEI/CD) NPs inhibited prostate cancer cell viability of PC-3 and DU-145. Furthermore, the poly(ethylene glycol)-poly(l-lactide) (PEG-PLA)-encapsulated AMG NPs ameliorated the neuropathology of Alzheimer’s disease by increasing the expression of low-density lipoprotein (LDLR) receptors in microglia and liver cells and accelerating clearance of amyloid-beta. In another study, oral administration of mucoadhesive NPs loaded with *Garcinia mangostana* extract eradicated *Helicobacter pylori* infection in mice [115].

## 6. Perspective

Nanotechnology has succeeded in increasing bioavailability and distribution, maintaining the integrity of compounds, increasing the biological activity of cancer drugs, and reducing the toxicity of conventional medicine [116]. Existing treatments against drug resistance have some limitations and fail to offer an adequate solution. Some of the mechanisms of breast-cancer resistance include (i) enzymes that can inactivate antitumor drugs, (ii) cancer-associated genes, (iii) cell membrane effects of drug absorption, (iv) DNA repair, and (v) tumor microenvironment. NPs can also overcome resistance to chemotherapy in breast cancer [117].

NPs DDS products are undoubtedly superior in therapeutic performance to conventional drug delivery systems and are therefore highly demanded [118,119,120]. Many nanoparticle surface structures have been developed for biomolecules and cell sensing, disease diagnosis, and intracellular delivery by adapting nanoparticle interfaces. Various types of ligands have been introduced to the surface of NPs [121].

NPs may increase selectivity, which is likely to reduce adverse side effects. Therefore, the NP formulation is an option to overcome the limitations of various drug compounds [106,107,108]. NP nanotechnology has been carried out to increase the solubility of AMG [105]. The increase in solubility occurs for the following reasons: (a) The reduction in particle size increases the surface area of the particles. (b) Adding excipients with high water solubility increases drug solubility through hydrogen bond interactions between excipients and water molecules [105,122,123]. NPs can be applied to controlled release, delayed-release, and continuous release systems [124,125]. The type of NP and the materials are adapted to the purpose and intended use [27,126,127].

In in vitro testing, exposure of cells to cytotoxic substances can result in cell death [53,54,55]. Various studies of AMG cause apoptosis [73,106,108,128]. The interaction of cancer cells with cytotoxic substances will also threaten the integrity of cell membranes and cause necrosis in a short time. The authors’ analysis showed that it is very likely for necrosis to occur, followed by apoptosis. Modifying cytotoxic substances’ solubility will increase exposure and significantly affect cytotoxicity and cancer cell death [82,129].

Research on AMG in vivo is still small compared to in vitro testing. In vitro testing requires a method that considers the mechanism of action of in vivo model molecules (51). Many attempts have been made to predict potential therapeutic candidates in vivo, and one such strategy is in vitro/in vivo correlation (IVIVC) [88]. In the future, IVIVC will be very important in pharmaceutical science to manufacture formulations and develop pharmaceutical preparations [130,131,132].

Marketed NPs have shown a better pharmacokinetic profile than free drugs. To achieve this goal, optimization aspects, such as nanoparticle size, shape, and surface charge, were carried out. The type and location of the tumor must be considered because of the characteristics of the specific microenvironment. Several approaches are now emerging to increase the permeability and penetration of particles within the tumor [133].

NPs show great potential for application in immunotherapy. The development of immunotherapy has brought cancer treatment into a new era by activating the antitumor immune response. NPs-associated immunotherapy includes nano-vaccines, artificial antigen-presenting cells, and targeting of the immune-suppressed tumor microenvironment (TME) [134]. The development of a vaccine induces antitumor immunity; HER2-protein-derived peptide, coupled with lambda phage (λF7) coat protein gpD, is potent against HER+ breast cancer in mice [135]. Immunotherapy has emerged as a BC treatment approach, especially TNBC, which is promising compared to other conventional modalities. Nanoparticle-based immunotherapy has a great potential to overcome these limitations. [133]. Selective targeting of tumor neovascularization components or targeting of tumor cells to enhance the immune system. Using antibodies to target negative immune modulators can provide a dual effect of the drug in NPs and specific antibodies. An example is the use of an anti-PD-L1 antibody (programmed death-ligand 1) that interferes with the inhibitory effect of the PD1/PD-L1 axis by acting on this receptor that is expressed in tumor cells. [133].

Multifunctional NPs will be a future research trend [59]. NPs can be modified through controlled synthesis, functionalization, or decoration by polymers [136]. Photothermal therapy (PTT) and photodynamic therapy (PDT) based on NPs have shown progression, small invasion, and mild side effects during tumor treatment [59]. In addition to directly killing tumor cells, the fragments of dead tumor cells produced by PDT and PTT treatment can be used as potential antigens to trigger a sustained immune response, called photothermal and photodynamic immunotherapy. As nanotechnology develops, it is necessary to monitor the immunotoxicity, long-term toxicity, and neurotoxicity of NPs. NPs would be an ideal approach to improve cancer therapy and diagnosis [137].

## 7. Conclusions

Plants containing secondary metabolites are the leading choice for cancer treatment, because they provide a safe alternative. AMG delivers a high value of cytotoxicity and cell death of cancer cells and its mechanism of action in inhibiting various stages of BC. This extraordinary antitumor potential makes AMG a promising drug for cancer treatment.

Nanotechnology has been widely exploited for superior cancer treatment, as compared to conventional drugs, such as increased stability and biocompatibility; it offers increased permeability and retention effect, and precise targeting. The use of nanotechnology in AMG gives a better impact than free compounds and has a promising potential in developing effective and efficient drug delivery for BC therapy. Nanotechnology improves chemotherapy and reduces its side effects by guiding drugs to target cancer cells selectively.

NPs are continuously being developed, developing toward a multifunctional drug delivery system by exploring properties and modifiable functional groups by adding ligands to actively target cancer cells. Surface-modified NP drug-delivery systems and active substances can be combined with diagnostic agents to form nano multifunction devices for therapeutic applications. Smart or intelligent drug delivery systems can deliver therapeutic molecules on-demand by altering the physicochemical properties. Stimuli-responsive drug-delivery systems have emerged as potential tools for the advanced treatment of cancers. These efforts will give great hope in the treatment of BC, with a combination of safe drugs and an effective and efficient delivery system. The next challenge is to hinder the design and development of NP drug delivery systems for clinical trials.

## Figures and Tables

**Table 1:** Pharmacological effects of AMG in vitro.

Anticancer Activity	Cell Models/Methods	Delivery	IC_50_/Concentration	Molecular Mechanisms	Reference
Cytotoxicity	SKBR3/The MTT assay/intracellular ROS level induced by H2 O2	AMG	8.21 μg/mL (ED50)	↓ cancer cell production	[70]
		Water-soluble extracts	160 μg/mL (ED50)	↓ cancer cell production	
	intracellular ROS level induced by H2O	AMG	20 μg/mL	↓ ROS	
		Water-soluble extracts	50 μg/ml	↓ ROS	
Attenuation of inflammation	MDA-MB-231		10 μM AMG −20 μM AMG	*iNOS protein ↓,* mRNA expressions COX-2↓, NO↓, PGE2production from PEG2↓	[71]
Induction of cell cycle arrest	MDA-MB-231 cells	12 μM–20 μM *AMG*	20 μM IC50 (24 h), 16 μM (48 h)	G1-arrest, p21CIP1expression↑, cyclins expression↓, CDC(s) expression↓, CDKs expression↓, PCNA↓, CHEK2 expression↑	[72]
Induction of the apoptotic signaling pathway	MDA-MB-231/The MTT assay	20 μM AMG	20 μM IC50 (24 h), 16 μM (48 h)	Mitochondrial membrane potential↓, caspase-7, caspase-8, caspase-9 and caspase-3 expression↑, ROS↑, Bcl-2 expression↓, Bax expression↑, Hsp70 protein expression↓, NF-κB translocation↓, PARP cleavage, Cytochrome c ↑	[72,73]
	MCF-7, MCF-10A(normal cell)/ The MTT assay	20 µg/mL AMG	IC50 10.26 ± 0.25 µg/mL (MCF-7), >0.30 µg/mL (MCF 10 A)		
	MCF-7 he MTT assay	1 mg/mL in DMSO of *AMG*	IC50 8–16 μM *AMG* after 24, under 6 μM *AMG not give cytotoxicity*	Caspases-8, caspase-9, and caspase−7 expressions↑, cytochrome c release, PARP cleavage, Bax expression↑, p53 expression↑, Bcl-2 expression↓, Bid↓, ERα expression↓	[74]
	MDA-MB, MCF 7/	24 h of exposure to AMG	4 μM AMG for 24 h, (IC50) value of 5.54 μM	Fatty acid synthase (FAS) ↓, ER stress↑, Autophagy inhibition ↑, LC3II/LC3I↑, p62 ↑	[74,75,76]
Inhibition of angiogenic and metastatic progression	Bovine retinal endothelial cells	1.4–8 μM AMG		VEGF-induced phosphorylation of VEGFR2 and ERK1/2-MAPK↓	[77]
	MCF-7/The MTT assay	20 µg/mL AMG	IC50 2 μM *AMG*	TNF-α-induced NF-κB translocation↓, NF-κB↓, c-Fos↓, and c-Jun↓	[74]

## Data Availability

Not applicable.
